# ADULT DAY SERVICES: THE OPTIMAL PLATFORM FOR GROUP INTERVENTION RESEARCH

**DOI:** 10.1093/geroni/igad104.0102

**Published:** 2023-12-21

**Authors:** Keith Anderson, Holly Dabelko-Schoeny

**Affiliations:** University of Mississippi, Oxford, Mississippi, United States; The Ohio State University, Columbus, Ohio, United States

## Abstract

Group intervention research with older adults is fraught with challenges related to access, recruitment, implementation, and sustainability. In this presentation we explore these challenges and offer adult day services (ADS) as an alternative platform to traditional long-term care sites (e.g., nursing homes – NHs, assisted living facilities – ALFs) for group intervention research. Access: NHs and ALFs are often reluctant to engage in research due to concerns with liability and fears that “outsiders” will expose deficits in their services. ADS programs are typically quite receptive to researchers and are eager to learn about innovative ways to improve their services. Recruitment: NHs and ALFs are comprised of individuals living in their private apartments and rooms and recruitment is often a one-on-one, time-consuming process. ADS programs are naturally collective environments where recruitment efforts can occur in a more efficient group format. Implementation: NHs and ALFs often lack adequate physical space and staff support for group research activities – key elements that contribute to successful implementation. ADS programs are specifically designed and staffed for group activities and interventions. Sustainability: The primary focus of NHs and ALFs is on “bed and body” care and long-term buy-in regarding group interventions and activities from facilities and staff is rare. Group programming is central to ADS and there is a much greater potential for buy-in and long-term sustainability. Conclusion: Researchers interested in group interventions should strongly consider ADS as the optimal platform and may find that the ADS setting offers the greatest potential for success.

